# Incorporating MXene into Boron Nitride/Poly(Vinyl Alcohol) Composite Films to Enhance Thermal and Mechanical Properties

**DOI:** 10.3390/polym13030379

**Published:** 2021-01-26

**Authors:** Seonmin Lee, Jooheon Kim

**Affiliations:** 1 School of Chemical Engineering & Materials Science, Chung-Ang University, Seoul 156-756, Korea; dltjsals7574@gmail.com; 2 Department of Intelligent Energy and Industry, Chung-Ang University, 84 Heukseok-ro, Dongjak-gu, Seoul 156-756, Korea

**Keywords:** composite film, MXene, boron nitride, thermal conductivity, surface modification

## Abstract

Aggregated boron nitride (ABN) is advantageous for increasing the packing and thermal conductivity of the matrix in composite materials, but can deteriorate the mechanical properties by breaking during processing. In addition, there are few studies on the use of Ti_3_C_2_ MXene as thermally conductive fillers. Herein, the development of a novel composite film is described. It incorporates MXene and ABN into poly(vinyl alcohol) (PVA) to achieve a high thermal conductivity. Polysilazane (PSZ)-coated ABN formed a heat conduction path in the composite film, and MXene supported it to further improve the thermal conductivity. The prepared polymer composite film is shown to provide through-plane and in-plane thermal conductivities of 1.51 and 4.28 W/mK at total filler contents of 44 wt.%. The composite film is also shown to exhibit a tensile strength of 11.96 MPa, which is much greater than that without MXene. Thus, it demonstrates that incorporating MXene as a thermally conductive filler can enhance the thermal and mechanical properties of composite films.

## 1. Introduction

In recent years, tremendous advances have been made in the field of electronics, including the development of small and highly-integrated light-emitting devices, printed circuit boards, and automotive parts. However, excessive heat generation in the device is increasingly a problem that results in device malfunction, substrate degradation, and performance degradation [1,2]. Therefore, thermally conductive materials are emerging as important components of electronic devices because they enable the efficient dissipation of heat in order to maintain the desired operating temperature. Although polymer-based composites have advantages in applications of heat dissipation, including light weight, low cost, facile processability, and electrical insulation [3,4,5], they are widely known to have low thermal conductivities (0.2–0.5 W/mK) and are not suitable for high-performance devices. As a result, various strategies have been employed to introduce thermally conductive fillers (including metal, ceramic, and carbon materials) into the polymer matrix to impart thermally conductive properties [6,7,8,9].

Boron nitride (BN) has attracted much attention as a thermally conductive filler material due to its electrical insulation, high thermal conductivity, and physicochemical stability [10,11,12,13,14]. The spherical shape of the BN particles promotes high packing densities and filler connectivities compared to other particles. Moreover, the spherical-shaped aggregated boron nitride (ABN) filler particles formed by the agglomeration of plate-shaped hexagonal BN (h-BN) provide a material that exhibits high thermal conductivity, excellent electrical insulation, high specific surface area, low viscosity, and excellent particle fluidity. Moreover, a previous study by the present authors established the capability of ABN for packing the matrix. Some researchers coated ABN with polysilazane (PSZ) to prevent the deformation of the ABN [15,16,17,18]. The function of the PSZ coating is to minimize the layer of air in the spherical ABN particles that results from the agglomeration of the h-BN particles, and thus prevent the agglomerates from breaking. For instance, Wie et al., coated ABN with PSZ and silane to protect it from breaking and improve the interfacial adhesion between the filler and the matrix [15]. The ABN modified with PSZ and aminopropyltriethoxysilane (APTES) exhibited a high thermal conductivity of 11.8 W/mK at a filler content of 75 wt.% in epoxy composite.

The MXenes are an emerging class of two-dimensional (2D) materials composed of transition metal carbides and/or carbonitrides with the general formula Mn + 1XnTx (where M is the transition metal, X is C and/or N, and Tx is the normal surface termination, which is an O, F or OH group). These materials exhibit unique two-dimensional layer structures and high specific surface areas, along with excellent thermal, adsorption, mechanical, and electrical properties. In particular, the Ti_3_C_2_ MXene exhibits inherently high metallic conductivity, tunability of surface composition via functional groups, and excellent mechanical stability. As a result, this material has been exploited in many fields such as energy storage, catalysis, adsorption, hydrogen storage, and sensing [19,20,21,22,23,24,25,26,27]. Nevertheless, few studies have reported the use of Ti_3_C_2_ MXene as a filler for improving the thermal conductivity of polymers. Thus, research into polymer composites that incorporate Ti_3_C_2_ MXene and/or other MXenes could have practical significance.

In this study, MXene is used as a thermally conductive filler to improve thermal conductivity and mechanical properties of a polymer composite with a ceramic filler. Poly(vinyl alcohol) (PVA) is used as the polymer matrix due to its excellent compatibility with MXene provided by the hydroxyl groups. Meanwhile, ABN is selected as the base material to support heat transfer from the PVA composite film. The ABN surface was modified with PSZ and hot pressed onto the PVA film to form a composite material. In addition, either (i) MXene is added to the PVA matrix as a second filler along with the PSZ-coated ABN, or (ii) the ABN surface is simultaneously coated with PSZ and MXene. Integration of MXenes into the composite is shown to improve the thermal conductivity and mechanical properties of the composite material, thus demonstrating the potential of MXenes as thermally conductive fillers.

## 2. Experimental Section

### 2.1. Materials

The ABN particles (average particle size of 50 μm) was obtained from LG Innotek (Seoul, Korea). PVA (MW 31,000~50,000, 98–99%) was supplied by Sigma-Aldrich (St. Louis, MO, USA). The KiON HTT1800 polysilazane (Clariant Specialty Chemicals, Pratteln, Switzerland) were used for surface modifications. MXene (98%) was purchased from Invisible (Gyeonggi-do, Korea).

### 2.2. Preparation of the Composite Film

**Figure 1:**
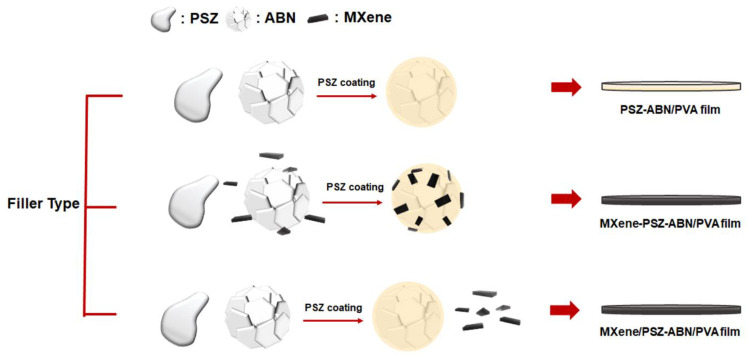
The schematic diagram of the composite fabrication routes.

The fabrication process for the MXene/PSZ-ABN composite films is shown schematically in Figure 1. In the present work, three composites were fabricated. The PSZ-coated ABN (designated PSZ-ABN), and the ABN coated with PSZ and MXene together (designated MXene-PSZ-ABN) were used as filler for the PVA matrix of the first and second composites, respectively. In the third composite (designated MXene/PSZ-ABN), MXene and PSZ-ABN were used as binary fillers. In each case, the thickness of the film was about 0.1 mm.

The MXenes were added to deionized (DI) water and subsequently sonicated. For the PSZ-ABN route, a mixture of PSZ and ABN was dispersed in DI water and stirred. Similarly, for the MXene-PSZ-ABN, MXene was stirred with PSZ and ABN in DI water. Each mixture was then heated to 90 °C with further stirring to remove the solvent. The remaining solute was moisture crosslinked at 160 °C for 12 h. The samples were gently ground during the first two hours to prevent clumping. Finally, the materials were sintered at 300 °C for two hours in a box furnace to yield the two types of filler material. Finally, the fillers were each dispersed in a PVA solution at 90 °C for two hours.

To make the third composite, PSZ-ABN and MXene were dispersed in PVA solution. The solution was added to a Teflon mold and a doctor blade method was used to obtain a film of uniform thickness. Processing was completed by hot pressing the film. Since MXene and PSZ-ABN were used as binary fillers in the third composite, MXene was randomly distributed among the ABN fillers.

The three composites are referred to as the PSZ-ABN/PVA film, the MXene-PSZ-ABN/PVA film, and the MXene/PSZ-ABN/PVA film (Figure 1). For simplicity, they are also referred to as the PSZ-ABN film, the MXene C film, and the MXene M film in the later Figures.

### 2.3. Characterization

The morphologies of the materials were observed by field-emission scanning electron microscopy (FE-SEM; Carl Zeiss, Oberkochen, Germany), and energy-dispersive X-ray spectroscopy (EDS) was used to map the surface chemical compositions. The X-ray diffraction (XRD; Bruker-AXS, D8-Advance) patterns were collected at a scan rate of 0.2°s^−1^ over a 2θ range of 10–80° with Cu Kα radiation (λ = 0.154056 nm). In addition, X-ray photoelectron spectroscopy (XPS; ESCA 2000 XPS, VG Microtech, London, UK) was used to analyze the PSZ and MXene coatings on the ABN composites. Thermogravimetric analysis (TGA) was performed using a CI Electronics microbalance (MK2-MC5) under an atmosphere of nitrogen gas. The samples were heated to 800 °C at a ramp rate of 10 °C/min, followed by an isothermal step at 800 °C for 30 min. The mechanical properties were measured using a universal testing machine (UTM; 3344Q9465, Instron Co., Norwood, MA, USA) at a crosshead speed rate of 5 mm/min. The thermal diffusivity was measured at room temperature via laser flash analysis (LFA; NanoFlash LFA 467, Netzsch Instruments Co., Selb, Germany), and the thermal conductivities were obtained using Equation (1):

*K* = *α ρ Cp* (1)

where *K* is the thermal conductivity, *ρ* is density, α is the thermal diffusivity, and *Cp* is the heat capacity of the composite. The density and heat capacities of each composite were calculated theoretically. The thermal conductivity enhancement (TCE) was calculated using Equation (2):

(2) $$\mathrm{TCE}=\frac{K-K_{m}}{K_{m}}\times 100\%\;$$

where K_m_ is the thermal conductivity of the matrix.

## 3. Results and Discussion

### 3.1. Synthesized Fillers Analysis

#### 3.1.1. XPS

**Figure 2:**
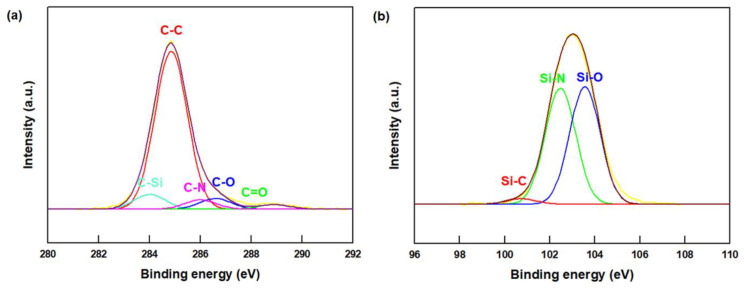
XPS analysis of the surface-modified aggregated boron nitride (ABN): (**a**) C 1s, (**b**) Si 2p deconvolution.

A detailed examination of the surface molecular structure of the PSZ-ABN is provided by the deconvoluted C 1s and Si 2p XPS spectra in Figure 2. Here, the peaks at 284.0, 284.8, 286.0, 286.6 and 288.9 eV in the C 1s spectrum are attributed to the C–Si, C–C, C–N, C–O and C=O bonds, respectively (Figure 2a), while the peaks at 102.5, 101.7, and 100.8 eV in the Si 2p spectrum are assigned to the Si–C, Si–N, and Si–O bonds, respectively (Figure 2b) [15,18]. These results demonstrate that the surface of the ABN particles was successfully modified by the treatment.

#### 3.1.2. Morphology

**Figure 3:**
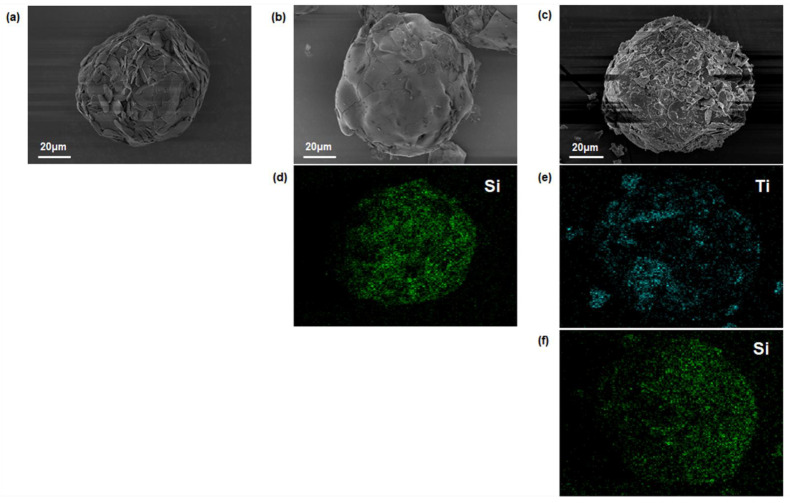
SEM image of surface modified ABN: (**a**) raw ABN, (**b**) polysilazane-coated ABN (PSZ-ABN), (**c**) MXene-PSZ-ABN. SEM-EDS mapping showing the distribution of (**d**) Si of PSZ-ABN, (**e**) Ti and (**f**) Si of MXene-PSZ-ABN.

The incorporation of MXene into the composite films is demonstrated by comparing the shapes of the raw ABN, the PSZ-ABN, and the MXene-PSZ-ABN revealed in the SEM images (Figure 3a–c). Thus, the presence of aggregated BN flakes is clearly evident on the rough surface of the raw ABN (Figure 3a), whereas the surface of the PSZ-ABN filler is smooth due to the absence of such aggregates (Figure 3b). Further, the incorporation of MXene into the MXene-PSZ-ABN particles is demonstrated by the appearance of additional surface features in Figure 3c.

The chemical natures of the raw ABN, PSZ-ABN, and MXene-PSZ-ABN surfaces are confirmed by the EDS mappings in Figure 3d–f. Here, the newly-formed material on the surface of the PSZ-ABN material is confirmed to be MXene, and the surface functionalization by PSZ is demonstrated. The surface of the raw ABN is rich in boron and nitrogen, whereas those of the PSZ-ABN and MXene-PSZ-ABN are covered with Si due to the presence of PSZ. In addition, the surface of the MXene-PSZ-ABN exhibits Ti due to the presence of MXene.

#### 3.1.3. Thermogravimetry

**Figure 4:**
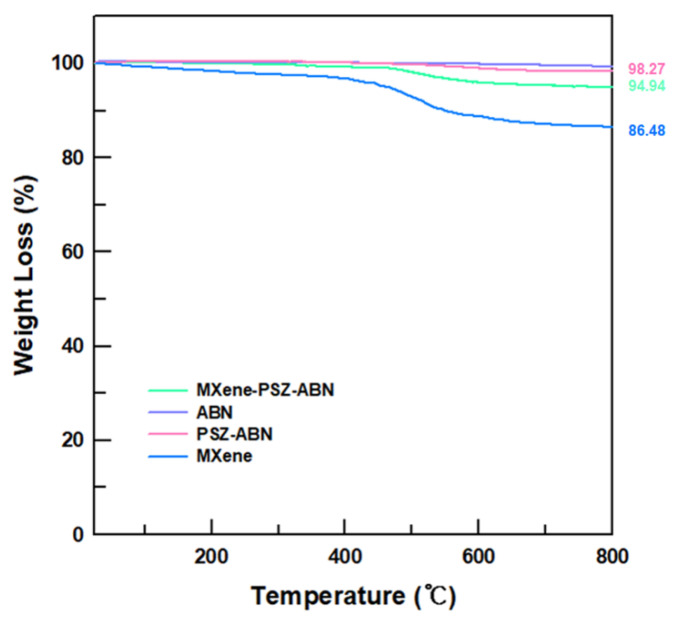
TGA curves of the raw ABN, the raw MXene, the PSZ-ABN, and the MXene-PSZ-ABN.

The quantities of the PSZ and MXene coatings were established by evaluating the ABN, PSZ-ABN, and MXene-PSZ-ABN fillers by TGA (Figure 4). Here, the raw-ABN exhibits almost no weight loss over the entire temperature range, thus demonstrating the high thermal stability of this material. By contrast, the weight loss of the MXene-coated sample between 25 and 200 °C is due to physically absorbed water and HF, while the weight loss above 200 °C is due to the removal of unstable chemically bonded -F, -OH, and -O groups that are present due to etching and ultrasonication [28]. However, the PSZ-ABN material exhibits almost no weight loss, which strongly implies that the physically attached PSZ was almost completely decomposed during the 300 °C sintering process [15]. Moreover, the difference in weight loss between the MXene-PSZ-ABN and the PSZ-ABN is 3.33%. Therefore, since the pristine MXene exhibits a weight loss of 13.52%, the MXene content of the MXene-PSZ-ABN is calculated to be about 25%. Thus, the TGA analyses of the MXene-PSZ-ABN materials provide clear evidence that the MXene is coated onto the PSZ-ABN surface.

### 3.2. Synthesized Composite Films Analysis

#### 3.2.1. Morphology

**Figure 5:**
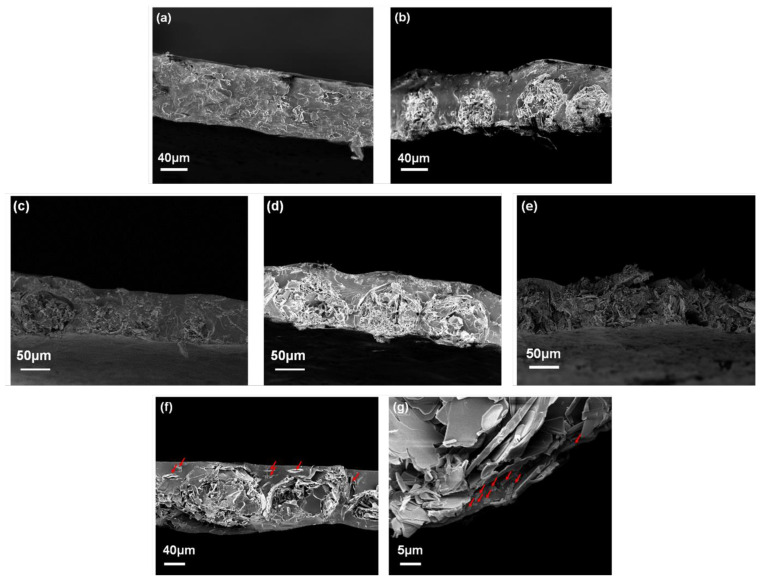
Cross-sectional SEM images of: (**a**) the ABN/PVA; (**b**) the PSZ-ABN/PVA; (**c–e**) the PSZ-ABN/PVA film with ABN loadings of 25 (**c**), 33 (**d**), and 40 wt.% (**e**); (**f**) MXene/PSZ-ABN/PVA film, and (**g**) the MXene-PSZ-ABN/PVA film. MXene is indicated by the red arrows.

The effect of the surface modified ABN with PSZ is shown in Figure 5a,b. PVA film packed with raw-ABN had the filler broken so that the shape of the ABN could not be recognized, whereas PSZ-ABN maintained the shape of the ABN well. When packing the polymer matrix of a certain thickness, it has been reported that the use of a filler having a size similar to that of the film provides advantages with respect to increasing the thermal conductivity [29]. It is suggested that the ABN particles form linear arrangements that can provide an effective pathway for heat transfer within the PVA film. To investigate this, composite films were also made with PSZ-ABN contents of 25, 33, and 40 wt.%. The film with 33 wt.% ABN provides the most desirable network of pathways for conduction (Figure 5d). The film with 25 wt.% ABN does not provide suitable pathways for heat conduction because the distance between the ABNs is too large (Figure 5c), while the addition of 40 wt.% ABN results in a nonuniform film thickness due to the excessive amount of ABN (Figure 5e). Thus, an ABN loading of 33 wt.% appears to be optimal and, hence, this optimal amount was used to prepare composite films in which the filler was well-incorporated into the PVA matrix, as demonstrated by the cross-sectional SEM images in Figure 5g,f. Note that the same amount of MXene was added to both the MXene-PSZ-ABN/PVA film and the MXene/PSZ-ABN/PVA film, the difference being that the ABN surface is surrounded by MXene and PSZ in the former (Figure 5g) whereas the PSZ-ABN is properly packed and the MXene is dispersed in the unpacked matrix in the latter (Figure 5f). In both images, the MXene is indicated by the red arrows.

#### 3.2.2. Mechanical Properties

**Figure 6:**
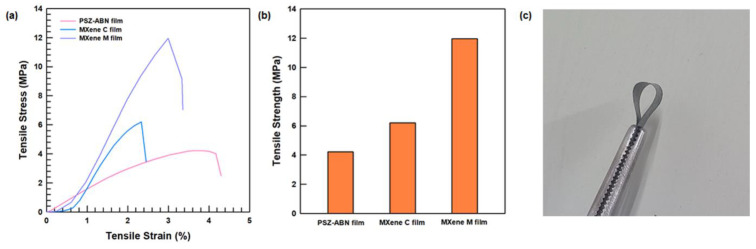
Mechanical properties of the composite films: (**a**) stress–strain curve, (**b**) tensile strength of PSZ-ABN film, MXene-PSZ-ABN (MXene C) film, and MXene/PSZ-ABN/PVA (MXene M) films; (**c**) a digital image of the folded MXene/PSZ-ABN/PVA film.

The stress-strain curves and tensile strengths of the PSZ-ABN, MXene-PSZ-ABN (MXene C), and MXene/PSZ-ABN/PVA (MXene M) composite films are presented in Figure 6. Here, the tensile strengths of the MXene-PSZ and MXene/PSZ-ABN/PVA composites are seen to have increased by 147% (to 6.20 MPa) and 283% (to 11.96 MPa), respectively, compared to that of the PSZ-ABN composite film in the absence of MXene (4.22 MPa). Despite the high loadings of filler promote poor mechanical properties, the composite material exhibited a strengthening effect as a result of adding MXene. This enhancement in mechanical properties, is related to the uniform dispersion of the filler and the hydrogen bonds that can form the -OH group between the MXene and the PVA matrix [30]. Thus, as demonstrated by the digital image in Figure 6c, the MXene/PSZ-ABN/PVA film retains the flexibility of PVA.

#### 3.2.3. Thermal Properties

**Figure 7:**
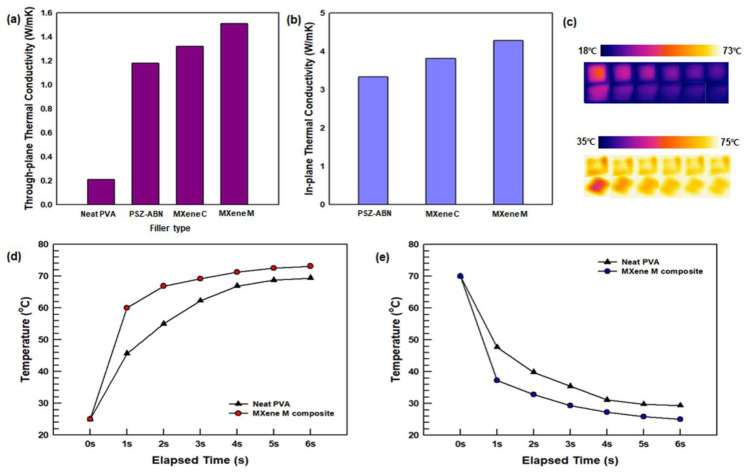
(**a**) Through-plane and (**b**) in-plane thermal conductivities of the composites; (**c**) infrared thermal images during heating and cooling; (**d**,**e**) the temperature-time curves obtained during heating (**d**) and cooling (**e**).

The through-plane and in-plane thermal conductivities of the neat PVA and the PSZ-ABN/PVA, MXene-PSZ-ABN/PVA, and MXene/PSZ-ABN/PVA composite films are presented in Figure 7. The thermal conductivity was measured by LFA methodology and Equation (1). Here, the film in which MXene is randomly distributed (i.e., MXene/PSZ-ABN/PVA) is seen to exhibit higher through-plane and in-plane thermal conductivities of 1.51 and 4.28 W/mK, respectively, compared to those of the MXene-PSZ-ABN (i.e., 1.32 and 3.81 W/mK, respectively). The TCEs of the MXene/PSZ-ABN/PVA and MXene-PSZ-ABN/PVA films are 719 and 629%, respectively. These results demonstrate that the thermal conductivities of the composites are improved by the addition of the MXene filler. This is explained by the good dispersion of the platelet-shaped MXene fillers in the matrix, which also promoted heat transfer in the in-plane direction due to their alignment in this direction according to their aspect ratio. Moreover, although the MXene-PSZ-ABN filler helped to improve the thermal conductivity of the PSZ-coating layer, it did not provide any further benefit in forming a thermal conduction pathway compared to the film in which the MXene was distributed in the matrix.

The thermal conduction performance of the neat PVA and the MXene/PSZ-ABN/PVA film is further demonstrated by the IR camera in Figure 7c. The graph in Figure 7d shows the temperature profiles obtained by placing neat PVA and the MXene/PS-ABN/PVA composite films on a hot plate and subjecting them to heating for 6 s. The temperature profiles of subsequent cooling processes are shown in Figure 7e. After 6 s of heating, the MXene/PSZ-ABN/PVA composites is seen to have reached a maximum temperature of 73.13 °C whereas the neat PVA has reached only 69.38 °C. Furthermore, the rates of temperature increase are seen to differ, with the MXene/PSZ-ABN/PVA reaching 60 °C in only one second whereas the neat PVA takes 3 s to reach this value. Similarly, the MXene/PSZ-ABN/PVA exhibited the faster cooling rate. Thus, the fabricated composite has much greater thermal conductivity than the neat PVA and is suitable for application as a thermally conductive material.

The MXene was expected to improve the thermal conductivity of the composite by connecting the ABN structure, which is the skeleton of the heat conduction network. As expected, the addition of MXene supplemented the existing heat transfer network to provide higher through-plane and in-plane thermal conductivities. In addition, the composite material exhibited excellent intrinsic mechanical properties along with a better tensile strength compared to that of the material without MXene. The present study has confirmed that the introduction of MXene as a thermally conductivity filler can have practical applicability. However, due to the high cost of MXene, future studies aimed at the easing the mass production process of composites using MXene will be needed.

## 4. Conclusions

A thermally conductive composite film was prepared consisting of PVA along with MXene and PSZ-coated ABN as fillers. The addition of MXene was shown to improve the thermal conductivity and mechanical properties of the resulting films by augmenting the thermal conductance of the PSZ-coated ABN filler. In detail, a composite with 44 wt.% filler was found to exhibit a through-plane thermal conductivity of 1.51 W/mK and the in-plane thermal conductivity of 4.28 W/mK. The composite film was also shown to be highly flexible and to exhibit a greater tensile strength of 11.96 MPa than that of the film made from PSZ-ABN alone (4.22 MPa). The present study has demonstrated that the thermal conductivity and mechanical properties of composite materials can be improved by incorporating MXene as a heat-conducting component. Such composites are suitable for application as thermal interface materials for electronic devices.

## Data Availability

The data presented in this study are available on request from the corresponding author.
